# Correction: Do we still need a canary in the coal mine for laboratory animal facilities? A systematic review of environmental health monitoring versus soiled bedding sentinels

**DOI:** 10.1371/journal.pone.0353658

**Published:** 2026-07-10

**Authors:** Megan R. LaFollette, Caroline S. Clement, Kerith R. Luchins, Christopher A. Manuel, Patricia L. Foley, Wai H. Hanson, Christina Pettan-Brewer, Caroline B. Winn, Joseph P. Garner

The acronym REM was defined incorrectly throughout the article. REM should be defined as “Room and Equipment Monitoring” rather than “Research Equipment Monitoring.”

As a result of the correction throughout the article, please see the correct Table 1 here.

**Table 1:** Common terminology for environmental health monitoring.

Environmental Health Monitoring (EHM)	A variety of diagnostic sampling methods used to indirectly or directly perform rodent colony health surveillance without the use of sentinel animals.
Exhaust Dust Testing (EDT)	The use of swabs or a media to collect dust and nucleic acid that accumulates in **primary housing equipment** such as an individually ventilated cage (IVC) exhaust system. Most commonly associated with exhaust air dust (EAD™) testing or exhaust debris testing (EDx) of housing systems lacking cage level filtration where the sample is representative of an entire housing unit such as a rack of IVC cages.
EDT: Plenum Swabbing	The use of swabs (flocked or sticky) to collect dust and nucleic acid from specific locations within the exhaust system of an IVC rack. This sample represents a snapshot in time based on the material that is present at collection.
EDT: In-line Media	The positioning of media in the exhaust air current which collects dust and nucleic acid through air turbulence. The media continuously collects material thus representing the entire duration of the sampling period.
Sentinel-Free Soiled Bedding (SFSB)	The serial pooling of soiled bedding from rodent colony cages which is then sampled by either swabs and/or media for particulates and nucleic acid. Procedurally resembles a SBS program and commonly used where static caging or IVC with cage level filtration of exhaust air occurs.
SFSB: Indwelling Media/Swabs	The pre-placement of swabs or media within a soiled bedding container which is then exposed to particulates and nucleic acid during the entire sampling period.
SFSB: Indwelling: Agitation	The intermittent shaking or stirring of pooled soiled bedding to help expose indwelling swabs or media to particulates and nucleic acid.
SFSB: Indwelling: Stagnant	The absence of any dedicated agitation or stirring action of pooled soil bedding to expose indwelling media or swabs to particulates and nucleic acid other than what occurs through routine handling and serial addition of material.
SFSB: Single Exposure: Dredging	Use of swabs and/or media that are dredged through serially pooled soiled bedding at the end of the collection period for a single exposure to particulates and nucleic acid.
Room and Equipment Monitoring (REM)	The use of swabs or media to sample husbandry or facility support equipment where dust and nucleic acid accumulate. Typically, the site of collection shows less precision in localizing the source and represents the health status of the animals within an entire housing room or facility.
Direct Colony Sampling (DCS)	The collection of feces, fur swabs, oral swabs, or blood to non-invasively (or minimally invasive) sample specific animals or their cage microenvironment. Typically used for quarantine testing of new arrival rodents or secondary/tertiary testing after a positive test by a different EHM method. This method is used due to the higher level of precision in pathogen localization at the cage level.
