# Supplementary material for: Do we still need a canary in the coal mine for laboratory animal facilities? A systematic review of environmental health monitoring versus soiled bedding sentinels
Source: PLoS One. 2024 Dec 5;19(12):e0311840. doi: 10.1371/journal.pone.0311840 (PMC11620448; doi:10.1371/journal.pone.0311840)
Supplement: S2 File — The study protocol written out a priori before beginning data collection. (PDF) [file pone.0311840.s002.pdf]

Environmental Health Monitoring for Rodents

Item #	Section/Subsection/Item	Description	Check for approval
	A. General		
1.	Title of the review	A systematic review of environmental health monitoring for laboratory mice and rats	
2.	Authors (names, affiliations, contributions)	Contribute to project management, creation of search strategy, management of data extraction, etc.:<br>• Megan R. LaFollette, MS, PhD, is Program Manager at the North American 3Rs Collaborative<br>• Joseph Garner, PhD, is a Professor at Stanford University<br>Contribute to article screening & data extraction:<br>• Caroline Clement, is an undergraduate student at Stanford University<br>Contribute to writing and reviewing the final manuscript:<br>• Theresa Cunningham-Faughnan MS, RLATG Assistant Professor, School of Health Professions and Nursing, Long Island University Brookville, NY<br>• Patricia L. Foley, DVM, DACLAM, Professor, Dept Microbiology and Immunology, Georgetown University<br>• Kerith R. Luchins, DVM, DACLAM, Director of Rodent Clinical Services at the the University of Chicago in Chicago, IL.<br>• Christopher A. Manuel, DVM, PhD, DACLAM, is the Senior Associate Directory of the Office of Laboratory Animal Resources at University of Colorado Anschutz Medical Campus in Aurora, CO.<br>• Christina Pettan-Brewer, DVM, MSc, is Teaching Professor and Co-Director of Comparative Medicine, School of Medicine at the University of Washington, Seattle, WA.<br>• Caroline Winn, DVM, MS, DACLAM is a Principal Scientist/Clinical Veterinarian at Comparative Medicine, Worldwide Research, Development and Medical, Pfizer Inc., Cambridge, MA.	
3.	Other contributors (names, affiliations, contributions)	Jane Yatcilla is a Veterinary Librarian at Purdue University, she contributed to the search strategy	
4.	Contact person + e-mail address	Megan LaFollette, meglafollette@na3rsc.org	
5.	Funding sources/sponsors	The North American 3Rs Collaborative	

6.	Conflicts of interest	Some funders of the North American 3Rs Collaborative sell products or services related to environmental monitoring or soiled bedding sentinels. However, none of these funders had any influence on the study objectives, data, or outcomes.	
7.	Date and location of protocol registration	2021-11-08 - SYRCLE	
8.	Registration number (if applicable)		
9.	Stage of review at time of registration	Conducting Search	
	B. Objectives		
	Background		
10.	What is already known about this disease/model/intervention? Why is it important to do this review?	Traditionally, live rodents are used as sentinels to monitor the health of rodent colonies. Commonly referred to as "Sentinels", these rodents receive soiled bedding from other rodents' cages with a goal of exposure to potential rodent infectious agents that may exist in the colony. Soiled bedding sentinels are the most common rodent health surveillance program type. However, increasing evidence indicates that using environmental health monitoring (EM) can be as or even more effective. In addition, EM does not use any live rodents to obtain the same information. EM uses molecular diagnostic tests which can detect miniscule amounts of genetic material of these infectious agents in the exhausted air or soiled bedding of colony cages. EM is touted as a possible replacement for soiled bedding sentinel programs. However, a recent survey indicates that relatively few institutions use EM although that is increasing. Having a centralized paper of evidence on the success and limitations of EM as compared to other live animal sentinel program may help increase the number of institutions that use EM, ultimately reducing the number of rodents used in research.	
	Research question		
11.	Specify the disease/health problem of interest	Viral, bacterial, and parasitic agents of mice and rats that have been shown to potentially impact animal health or research study outcomes.	
12.	Specify the population/species studied	Mice or Rats	
13.	Specify the intervention/exposure	Environmental Health Monitoring (case studies are allowed), PCR analysis of environmental samples.	
14.	Specify the control population	Sentinel rodents (not restricted to controlled studies)	
15.	Specify the outcome measures	Detection Accuracy, Cost of EM (PCR vs. serology, cost of animals, per diems, labor), agent detection, comparison of EM to soiled bedding sentinels	
16.	State your research question (based on items 11-15)	Primary: What infectious agents can be reliably detected for mice or rats via environmental health monitoring?<br>Secondary: Of the infectious agents investigated, is EM better at detecting (more sensitivity and specificity)	

		infectious agents as compared to live animal surveillance programs.	
	C. Methods		
	Search and study identification		
17.	Identify literature databases to search (*e.g.* Pubmed, Embase, Web of science)	X MEDLINE via PubMed X Web of Science<br>X SCOPUS ☐ EMBASE<br>☐ Other, namely: CAAB<br>☐ Specific journal(s), namely:	
18.	Define electronic search strategies (*e.g.* use the step by step search guide[15] and animal search filters[20, 21])	When available, please add a supplementary file containing your search strategy: [insert file name]	
19.	Identify other sources for study identification	X Reference lists of included studies ☐ Books<br>X Reference lists of relevant reviews<br>X Conference proceedings, namely: American Association for Laboratory Animal Science [https://www.aalas.org/national-meeting/abstract-archive]<br>☐ Contacting authors/ organisations, namely:<br>☐ Other, namely:	
20.	Define search strategy for these other sources	We will scan the reference lists of included studies and relevant reviews for other relevent studies that were not captured by our search. We will search AALAS conference proceedings from the date of the first publication on EM to the present.	
	Study selection		
21.	Define screening phases (*e.g.* pre-screening based on title/abstract, full text screening, both)	1. Pre-screening = title/abstract<br>2. Full text screening	
22.	Specify (a) the number of reviewers per screening phase and (b) how discrepancies will be resolved	(a) 1 reviewer code all (Undergraduate assistant) and 1 reviewer code 20% (additional undergraduate or member of our environmental monitoring initiative)<br>(b) Disagreements will be resolved via discussion and consultation with a third party	
	Define all inclusion and exclusion criteria based on:		
23.	Type of study (design)	Inclusion criteria: experimental study or case report<br>Exclusion criteria: any other study design (e.g., reviews)	
24.	Type of animals/population (*e.g.* age, gender, disease model)	Inclusion criteria: mice or rats<br>Exclusion criteria: other species	
25.	Type of intervention (*e.g.* dosage, timing, frequency)	Inclusion criteria: use some sort of environmental health monitoring (e.g., a variety of sampling methods that are used to indirectly perform rodent colony health surveillance via PCR testing (e.g., exhaust air debris/IVC monitoring)<br>Exclusion criteria: no use of environmental monitoring	
26.	Outcome measures	Inclusion criteria: factors relevant to testing to viral, bacterial, and parasitic agents of mice and rats (e.g.	

		detection ability or accuracy, comparison to soiled bedding sentinels) Cost of EM (PCR vs. serology, cost of animals, per diems, labor)<br>Exclusion criteria: non-relevant outcome measures	
27.	Language restrictions	Inclusion criteria: studies written in English<br>Exclusion criteria: Any language other than English	
28.	Publication date restrictions	Inclusion criteria:<br>Exclusion criteria:	
29.	Other	Inclusion criteria:<br>Exclusion criteria: None	
30.	Sort and prioritize your exclusion criteria per selection phase	Selection phase: Pre-screening<br>1. Exclude if not in English<br>2. Exclude if review<br>3. Exclude if not Mice or rats<br>4. Exclude if not Environmental health monitoring<br>5. Exclude if no relevant outcome measures<br><br>Selection phase: Full-text<br>1. Exclude if not in English<br>2. Exclude if review<br>3. Exclude if not Mice or rats<br>4. Exclude if not Environmental health monitoring<br>5. Exclude if no relevant outcome measures	
	Study characteristics to be extracted (for assessment of external validity, reporting quality)		
31.	Study ID (*e.g.* authors, year)	Author, Year, Title, Journal Name	
32.	Study design characteristics (*e.g.* experimental groups, number of animals)	Study type: Case report, Experimental<br># of comparisons<br># of facilities<br># of cages<br>Length of monitoring period<br>Type of sampling: environmental health monitoring as a complete replacement or as an additional tool<br>Type of caging: Rack level filtration vs Cage level filtration ((May be inferred by recording IVC Rack Manufacturer (Allentown, Tecniplast, Lab Products, Animal Care Systems, Thoren, Innovive, etc.))<br>Frequency of health monitoring performed<br>Air Changes per Hour of the IVC<br>Sanitization practice of the racks<br>Media type or swab used for genomic material collection	
33.	Animal model characteristics (*e.g.* species, gender, disease induction)	Species<br>Strain	
34.	Intervention characteristics (*e.g.* intervention, timing, duration)	Method of detection: plenum swabs, filters, sticky swabs, shaking, etc.<br>Pathogens tested for<br>Duration and frequency of sampling	
35.	Outcome measures	Detection, Money, time, and Labor spent	
36.	Other (*e.g.* drop-outs)		
	Assessment risk of bias (internal validity) or study quality		
37.	Specify (a) the number of reviewers assessing the risk of bias/study quality	(a) 1 main reviewer & 1 code 20% using collectively predefined assessment criteria.	

	in each study and (b) how discrepancies will be resolved	(b) Discrepancy resolved through discussion	
38.	Define criteria to assess (a) the internal validity of included studies (*e.g.* selection, performance, detection and attrition bias) and/or (b) other study quality measures (*e.g.* reporting quality, power)	☐By use of SYRCLE's Risk of Bias tool[4]<br>XBy use of SYRCLE's Risk of Bias tool, adapted as follows: Add "not applicable" as a possible answer to accommodate the use of this tool to evaluate a wide variety of experimental designs<br>☐By use of CAMARADES' study quality checklist, e.g [22]<br>☐By use of CAMARADES' study quality checklist, adapted as follows:<br>☐Other criteria, namely:	
	Collection of outcome data		
39.	For each outcome measure, define the type of data to be extracted (*e.g.* continuous/dichotomous, unit of measurement)	Continuous number, unit of measurement, etc.	
40.	Methods for data extraction/retrieval (*e.g.* first extraction from graphs using a digital screen ruler, then contacting authors)	From text or tables, then contacting authors	
41.	Specify (a) the number of reviewers extracting data and (b) how discrepancies will be resolved	1 reviewer, 2nd reviewer check random 20%	
	Data analysis/synthesis		
42.	Specify (per outcome measure) how you are planning to combine/compare the data (*e.g.* descriptive summary, meta-analysis)	# of studies successfully detecting each pathogen or not	
43.	Specify (per outcome measure) how it will be decided whether a meta-analysis will be performed	No meta-analysis	
	If a meta-analysis seems feasible/sensible, specify (for each outcome measure):		
44.	The effect measure to be used (*e.g.* mean difference, standardized mean difference, risk ratio, odds ratio)	Not applicable	
45.	The statistical model of analysis (*e.g.* random or fixed effects model)	Not applicable	
46.	The statistical methods to assess heterogeneity (*e.g.* I^2, Q)	Not applicable	
47.	Which study characteristics will be examined as potential source of heterogeneity (subgroup analysis)	Not applicable	
48.	Any sensitivity analyses you propose to perform	Not applicable	
49.	Other details meta-analysis (*e.g.* correction for multiple testing, correction for multiple use of control group)	Not applicable	
50.	The method for assessment of publication bias	Not applicable	

Final approval by (names, affiliations):		Date:
Megan LaFollette, The North American 3Rs Collaborative		Nov 8, 2021
