# Supplementary material for: Do we still need a canary in the coal mine for laboratory animal facilities? A systematic review of environmental health monitoring versus soiled bedding sentinels
Source: PLoS One. 2024 Dec 5;19(12):e0311840. doi: 10.1371/journal.pone.0311840 (PMC11620448; doi:10.1371/journal.pone.0311840)
Supplement: S3 File — The full text search strategy used for each database. (DOCX) [file pone.0311840.s003.docx]

PubMed:

1. (Biological Monitoring[MESH]) **750**
2. ((environmental monitoring[Title/Abstract]) OR (health monitoring[Title/Abstract]) OR (hygienic monitoring[Title/Abstract]) OR (health surveillance[Title/Abstract]) OR (microbiological monitoring[Title/Abstract]) OR (diagnostic surveillance[Title/Abstract]) OR (routine surveillance[Title/Abstract]) OR (sampling bedding debris [Title/Abstract]) OR (Hygienic surveillance[Title/Abstract]) OR (environmental sampling[Title/Abstract])) **21,075**
3. **(**(exhaust air[Title/Abstract]) OR (exhaust debris[Title/Abstract])) OR (exhaust dust[Title/Abstract])) **247**
4. 1 OR 2 OR 3 **22,035**
5. ((Murinae[MeSH Terms]) OR (mice[Title/Abstract] OR rats[Title/Abstract])) **3,355,817**
6. 4 AND 5 **330**
7. 6 AND English **320 - 386**

Web of Science:

1. MESH terms not used in Web of Science
2. TS=((("environmental monitoring") OR ("health monitoring") OR ("hygienic monitoring") OR ("health surveillance") OR ("microbiological monitoring") OR ("diagnostic surveillance") OR ("routine surveillance") OR ("sampling of bedding debris") OR ("Hygienic surveillance") OR ("environmental sampling"))) **65,030**
3. TS=(((("exhaust air") OR ("exhaust debris")) OR ("exhaust dust"))) **1,604**
4. 2 OR 3 **66,612**
5. TS=(((Murinae) OR (mice OR rats))) **3,422,118**
6. 4 AND 5 **471**
7. 6 AND English **456**

CAB Abstracts:

1. MESH terms not used in CAB Abstracts
2. title:((("environmental monitoring") OR ("health monitoring") OR ("hygienic monitoring") OR ("health surveillance") OR ("microbiological monitoring") OR ("diagnostic surveillance") OR ("routine surveillance") OR ("sampling of bedding debris") OR ("Hygienic surveillance") OR ("environmental sampling"))) OR ab:((("environmental monitoring") OR ("health monitoring") OR ("hygienic monitoring") OR ("health surveillance") OR ("microbiological monitoring") OR ("diagnostic surveillance") OR ("routine surveillance") OR ("sampling of bedding debris") OR ("Hygienic surveillance") OR ("environmental sampling"))) **18,420**
3. title:(((("exhaust air") OR ("exhaust debris")) OR ("exhaust dust"))) OR ab:(((("exhaust air") OR ("exhaust debris")) OR ("exhaust dust"))) **747**
4. 2 OR 3 **19,150**
5. title:(((Murinae) OR (mice OR rats))) OR ab:(((Murinae) OR (mice OR rats)))
6. 4 AND 5 **325**
7. 6 AND English **295**
8. 7 AND journal article **285**
