# Supplementary material for: Do we still need a canary in the coal mine for laboratory animal facilities? A systematic review of environmental health monitoring versus soiled bedding sentinels
Source: PLoS One. 2024 Dec 5;19(12):e0311840. doi: 10.1371/journal.pone.0311840 (PMC11620448; doi:10.1371/journal.pone.0311840)
Supplement: S2 Dataset — This file includes the SAS code for the matched dataset only including the publications that directly compared EHM and SBS and the corresponding analyses and steps. (DOCX) [file pone.0311840.s008.docx]

/* Dataset trimmed to include only articles with a "matched" design that included SBS as a control */

DATA EHMSysRevMatch20240117; INPUT PathogenByType &$15. PathogenByImportance &$14. Article &$18. Sampling_Method &$ EHMvsSBS &$ Detected_YesNo &$; Lines;

Virus Optional Compton 2004 SBS SBS No

Virus Optional Compton 2004 EDT EHM No

Virus 3MonthorAnnual Compton 2004 SBS SBS No

Endoparasite Optional Dubelko 2018 SBS SBS No

Ectoparasites 3MonthorAnnual Hanson 2021 SBS SBS No

BacteriaorFungi 3MonthorAnnual Kim 2022 SBS SBS No

BacteriaorFungi Optional Kim 2022 DCS EHM No

Ectoparasites 3MonthorAnnual Korner 2019 SBS SBS No

BacteriaorFungi 3MonthorAnnual Miller 2016 SBS SBS No

Ectoparasites 3MonthorAnnual Miller 2018 SBS SBS No

Endoparasite Optional Miller 2018 SBS SBS No

Endoparasite 3MonthorAnnual Miller 2018 SBS SBS No

BacteriaorFungi Optional Miller 2018 SBS SBS No

BacteriaorFungi Optional Miller 2018 SBS SBS No

BacteriaorFungi Optional Miller 2018 EDT EHM No

BacteriaorFungi 3MonthorAnnual Miller 2018 SBS SBS No

BacteriaorFungi Optional Niimi 2018 SBS SBS No

BacteriaorFungi 3MonthorAnnual Niimi 2018 SBS SBS No

Endoparasite Optional Niimi 2018 SBS SBS No

BacteriaorFungi 3MonthorAnnual O'Connell 2021 SBS SBS No

BacteriaorFungi Optional Schlapp 2018 SBS SBS No

BacteriaorFungi Optional Schlapp 2018 SBS SBS No

BacteriaorFungi Optional Tierce 2022 SBS SBS No

Ectoparasites 3MonthorAnnual Varela 2022 SBS SBS No

BacteriaorFungi Optional Winn 2022 DCS EHM No

BacteriaorFungi Optional Winn 2022 DCS EHM No

BacteriaorFungi Optional Winn 2022 SFSB EHM No

BacteriaorFungi Optional Winn 2022 DCS EHM No

BacteriaorFungi 3MonthorAnnual Bucheister 2020 SBS SBS Yes

BacteriaorFungi 3MonthorAnnual Bucheister 2020 EDT EHM Yes

BacteriaorFungi 3MonthorAnnual Compton 2004 SBS SBS Yes

BacteriaorFungi 3MonthorAnnual Compton 2004 EDT EHM Yes

Virus 3MonthorAnnual Compton 2004 SBS SBS Yes

Virus 3MonthorAnnual Compton 2004 EDT EHM Yes

Virus 3MonthorAnnual Compton 2004 SBS SBS Yes

Virus 3MonthorAnnual Compton 2004 EDT EHM Yes

Virus 3MonthorAnnual Compton 2004 EDT EHM Yes

Virus Optional Compton 2017 SBS SBS Yes

Virus Optional Compton 2017 EDT EHM Yes

Virus Optional Compton 2017 DCS EHM Yes

Endoparasite Optional Dubelko 2018 SBS SBS Yes

Endoparasite Optional Dubelko 2018 SFSB EHM Yes

Endoparasite Optional Dubelko 2018 DCS EHM Yes

BacteriaorFungi 3MonthorAnnual Dubelko 2018 SBS SBS Yes

BacteriaorFungi 3MonthorAnnual Dubelko 2018 SFSB EHM Yes

BacteriaorFungi 3MonthorAnnual Dubelko 2018 DCS EHM Yes

Virus 3MonthorAnnual Dubelko 2018 SBS SBS Yes

Virus 3MonthorAnnual Dubelko 2018 SFSB EHM Yes

Virus 3MonthorAnnual Dubelko 2018 DCS EHM Yes

BacteriaorFungi 3MonthorAnnual Dubelko 2018 SBS SBS Yes

BacteriaorFungi 3MonthorAnnual Dubelko 2018 SFSB EHM Yes

BacteriaorFungi 3MonthorAnnual Dubelko 2018 DCS EHM Yes

Endoparasite Optional Dubelko 2018 SFSB EHM Yes

Endoparasite Optional Dubelko 2018 DCS EHM Yes

Ectoparasites 3MonthorAnnual Gerwin 2017 SBS SBS Yes

Ectoparasites 3MonthorAnnual Gerwin 2017 SFSB EHM Yes

Endoparasite 3MonthorAnnual Gerwin 2017 SBS SBS Yes

Endoparasite 3MonthorAnnual Gerwin 2017 SFSB EHM Yes

Ectoparasites 3MonthorAnnual Hanson 2021 SFSB EHM Yes

BacteriaorFungi 3MonthorAnnual Hanson 2021 SBS SBS Yes

BacteriaorFungi 3MonthorAnnual Hanson 2021 SFSB EHM Yes

Virus 3MonthorAnnual Hanson 2021 SBS SBS Yes

Virus 3MonthorAnnual Hanson 2021 SFSB EHM Yes

BacteriaorFungi 3MonthorAnnual Kim 2022 EDT EHM Yes

BacteriaorFungi 3MonthorAnnual Kim 2022 DCS EHM Yes

BacteriaorFungi Optional Kim 2022 SBS SBS Yes

BacteriaorFungi Optional Kim 2022 EDT EHM Yes

Ectoparasites 3MonthorAnnual Korner 2019 EDT EHM Yes

Virus Optional Korner 2019 SBS SBS Yes

Virus Optional Korner 2019 EDT EHM Yes

Endoparasite 3MonthorAnnual Leblanc 2014 SBS SBS Yes

Endoparasite 3MonthorAnnual Leblanc 2014 DCS EHM Yes

BacteriaorFungi 3MonthorAnnual Mailhiot 2020 SBS SBS Yes

BacteriaorFungi 3MonthorAnnual Mailhiot 2020 EDT EHM Yes

Virus 3MonthorAnnual Mailhiot 2020 SBS SBS Yes

Virus 3MonthorAnnual Mailhiot 2020 EDT EHM Yes

BacteriaorFungi 3MonthorAnnual Mailhiot 2020 SBS SBS Yes

BacteriaorFungi 3MonthorAnnual Mailhiot 2020 EDT EHM Yes

BacteriaorFungi 3MonthorAnnual Miller 2016 EDT EHM Yes

Ectoparasites 3MonthorAnnual Miller 2018 EDT EHM Yes

Endoparasite Optional Miller 2018 EDT EHM Yes

BacteriaorFungi 3MonthorAnnual Miller 2018 SBS SBS Yes

BacteriaorFungi 3MonthorAnnual Miller 2018 EDT EHM Yes

BacteriaorFungi Optional Miller 2018 SBS SBS Yes

BacteriaorFungi Optional Miller 2018 EDT EHM Yes

Virus 3MonthorAnnual Miller 2018 SBS SBS Yes

Virus 3MonthorAnnual Miller 2018 EDT EHM Yes

Endoparasite 3MonthorAnnual Miller 2018 EDT EHM Yes

BacteriaorFungi Optional Miller 2018 EDT EHM Yes

BacteriaorFungi Optional Miller 2018 EDT EHM Yes

BacteriaorFungi Optional Miller 2018 SBS SBS Yes

BacteriaorFungi 3MonthorAnnual Miller 2018 EDT EHM Yes

BacteriaorFungi Optional Miller 2018 SBS SBS Yes

BacteriaorFungi Optional Miller 2018 EDT EHM Yes

Endoparasite Optional Miller 2018 SBS SBS Yes

Endoparasite Optional Miller 2018 EDT EHM Yes

BacteriaorFungi 3MonthorAnnual Neubert 2022 SBS SBS Yes

BacteriaorFungi 3MonthorAnnual Neubert 2022 DCS EHM Yes

BacteriaorFungi 3MonthorAnnual Neubert 2022 SBS SBS Yes

BacteriaorFungi 3MonthorAnnual Neubert 2022 DCS EHM Yes

BacteriaorFungi 3MonthorAnnual Neubert 2022 SBS SBS Yes

BacteriaorFungi 3MonthorAnnual Neubert 2022 DCS EHM Yes

BacteriaorFungi 3MonthorAnnual Neubert 2022 SBS SBS Yes

BacteriaorFungi 3MonthorAnnual Neubert 2022 DCS EHM Yes

BacteriaorFungi 3MonthorAnnual Neubert 2022 SBS SBS Yes

BacteriaorFungi 3MonthorAnnual Neubert 2022 DCS EHM Yes

Ectoparasites 3MonthorAnnual Niimi 2018 SBS SBS Yes

Ectoparasites 3MonthorAnnual Niimi 2018 EDT EHM Yes

Endoparasite Optional Niimi 2018 SBS SBS Yes

Endoparasite Optional Niimi 2018 EDT EHM Yes

Endoparasite Optional Niimi 2018 DCS EHM Yes

BacteriaorFungi 3MonthorAnnual Niimi 2018 SBS SBS Yes

BacteriaorFungi 3MonthorAnnual Niimi 2018 EDT EHM Yes

BacteriaorFungi 3MonthorAnnual Niimi 2018 DCS EHM Yes

BacteriaorFungi Optional Niimi 2018 EDT EHM Yes

BacteriaorFungi 3MonthorAnnual Niimi 2018 EDT EHM Yes

BacteriaorFungi 3MonthorAnnual Niimi 2018 DCS EHM Yes

BacteriaorFungi Optional Niimi 2018 SBS SBS Yes

BacteriaorFungi Optional Niimi 2018 EDT EHM Yes

BacteriaorFungi Optional Niimi 2018 DCS EHM Yes

Endoparasite Optional Niimi 2018 EDT EHM Yes

Endoparasite Optional Niimi 2018 DCS EHM Yes

BacteriaorFungi 3MonthorAnnual O'Connell 2021 SFSB EHM Yes

Virus 3MonthorAnnual O'Connell 2021 SBS SBS Yes

Virus 3MonthorAnnual O'Connell 2021 SFSB EHM Yes

Virus 3MonthorAnnual O'Connell 2021 SBS SBS Yes

Virus 3MonthorAnnual O'Connell 2021 SFSB EHM Yes

Virus 3MonthorAnnual O'Connell 2021 SBS SBS Yes

Virus 3MonthorAnnual O'Connell 2021 SFSB EHM Yes

Virus Optional O'Connell 2021 SBS SBS Yes

Virus Optional O'Connell 2021 SFSB EHM Yes

Endoparasite 3MonthorAnnual O'Connell 2021 SBS SBS Yes

Endoparasite 3MonthorAnnual O'Connell 2021 SFSB EHM Yes

Virus 3MonthorAnnual O'Connell 2021 SBS SBS Yes

Virus 3MonthorAnnual O'Connell 2021 SFSB EHM Yes

BacteriaorFungi Optional Scavizzi 2021 SBS SBS Yes

BacteriaorFungi Optional Scavizzi 2021 DCS EHM Yes

BacteriaorFungi 3MonthorAnnual Scavizzi 2021 SBS SBS Yes

BacteriaorFungi 3MonthorAnnual Scavizzi 2021 DCS EHM Yes

Ectoparasites 3MonthorAnnual Scavizzi 2021 SBS SBS Yes

Ectoparasites 3MonthorAnnual Scavizzi 2021 DCS EHM Yes

Endoparasite Optional Scavizzi 2021 SBS SBS Yes

Endoparasite Optional Scavizzi 2021 DCS EHM Yes

BacteriaorFungi 3MonthorAnnual Scavizzi 2021 SBS SBS Yes

BacteriaorFungi 3MonthorAnnual Scavizzi 2021 DCS EHM Yes

Virus 3MonthorAnnual Scavizzi 2021 SBS SBS Yes

Virus 3MonthorAnnual Scavizzi 2021 DCS EHM Yes

Endoparasite 3MonthorAnnual Scavizzi 2021 SBS SBS Yes

Endoparasite 3MonthorAnnual Scavizzi 2021 DCS EHM Yes

BacteriaorFungi 3MonthorAnnual Scavizzi 2021 SBS SBS Yes

BacteriaorFungi 3MonthorAnnual Scavizzi 2021 DCS EHM Yes

Endoparasite Optional Scavizzi 2021 SBS SBS Yes

Endoparasite Optional Scavizzi 2021 DCS EHM Yes

BacteriaorFungi Optional Scavizzi 2021 SBS SBS Yes

BacteriaorFungi Optional Scavizzi 2021 DCS EHM Yes

BacteriaorFungi 3MonthorAnnual Scavizzi 2021 SBS SBS Yes

BacteriaorFungi 3MonthorAnnual Scavizzi 2021 DCS EHM Yes

Endoparasite Optional Scavizzi 2021 SBS SBS Yes

Endoparasite Optional Scavizzi 2021 DCS EHM Yes

BacteriaorFungi Optional Schlapp 2018 EDT EHM Yes

BacteriaorFungi Optional Schlapp 2018 EDT EHM Yes

BacteriaorFungi Optional Tierce 2022 DCS EHM Yes

Ectoparasites 3MonthorAnnual Varela 2022 EDT EHM Yes

Ectoparasites 3MonthorAnnual Varela 2022 SFSB EHM Yes

Ectoparasites 3MonthorAnnual Varela 2022 DCS EHM Yes

Endoparasite Optional Varela 2022 SBS SBS Yes

Endoparasite Optional Varela 2022 EDT EHM Yes

Endoparasite Optional Varela 2022 SFSB EHM Yes

Endoparasite Optional Varela 2022 DCS EHM Yes

BacteriaorFungi 3MonthorAnnual Varela 2022 SBS SBS Yes

BacteriaorFungi 3MonthorAnnual Varela 2022 EDT EHM Yes

BacteriaorFungi 3MonthorAnnual Varela 2022 SFSB EHM Yes

BacteriaorFungi 3MonthorAnnual Varela 2022 DCS EHM Yes

Virus 3MonthorAnnual Varela 2022 SBS SBS Yes

Virus 3MonthorAnnual Varela 2022 EDT EHM Yes

Virus 3MonthorAnnual Varela 2022 SFSB EHM Yes

Virus 3MonthorAnnual Varela 2022 DCS EHM Yes

Virus 3MonthorAnnual Varela 2022 SBS SBS Yes

Virus 3MonthorAnnual Varela 2022 EDT EHM Yes

Virus 3MonthorAnnual Varela 2022 SFSB EHM Yes

Virus 3MonthorAnnual Varela 2022 DCS EHM Yes

BacteriaorFungi Optional Varela 2022 SBS SBS Yes

BacteriaorFungi Optional Varela 2022 EDT EHM Yes

BacteriaorFungi Optional Varela 2022 SFSB EHM Yes

BacteriaorFungi Optional Varela 2022 DCS EHM Yes

BacteriaorFungi 3MonthorAnnual Varela 2022 SBS SBS Yes

BacteriaorFungi 3MonthorAnnual Varela 2022 EDT EHM Yes

BacteriaorFungi 3MonthorAnnual Varela 2022 SFSB EHM Yes

BacteriaorFungi 3MonthorAnnual Varela 2022 DCS EHM Yes

Endoparasite Optional Varela 2022 SBS SBS Yes

Endoparasite Optional Varela 2022 EDT EHM Yes

Endoparasite Optional Varela 2022 SFSB EHM Yes

Endoparasite Optional Varela 2022 DCS EHM Yes

BacteriaorFungi Optional Varela 2022 SBS SBS Yes

BacteriaorFungi Optional Varela 2022 EDT EHM Yes

BacteriaorFungi Optional Varela 2022 SFSB EHM Yes

BacteriaorFungi Optional Varela 2022 DCS EHM Yes

Endoparasite Optional Varela 2022 SBS SBS Yes

Endoparasite Optional Varela 2022 EDT EHM Yes

Endoparasite Optional Varela 2022 SFSB EHM Yes

Endoparasite Optional Varela 2022 DCS EHM Yes

BacteriaorFungi Optional Winn 2022 SBS SBS Yes

BacteriaorFungi Optional Winn 2022 SFSB EHM Yes

Virus Optional Winn 2022 SBS SBS Yes

Virus Optional Winn 2022 SFSB EHM Yes

Virus Optional Winn 2022 DCS EHM Yes

BacteriaorFungi Optional Winn 2022 SBS SBS Yes

BacteriaorFungi Optional Winn 2022 SFSB EHM Yes

BacteriaorFungi Optional Winn 2022 SBS SBS Yes

BacteriaorFungi Optional Winn 2022 SFSB EHM Yes

BacteriaorFungi Optional Winn 2022 DCS EHM Yes

BacteriaorFungi Optional Winn 2022 SBS SBS Yes

Virus 3MonthorAnnual Zorn 2016 SBS SBS Yes

Virus 3MonthorAnnual Zorn 2016 EDT EHM Yes

;

/*Pathogen by Type Analysis */

PROC GENMOD DATA=EHMSysRevMatch20240117 DESC;

/* The DESC sorting option ensures that the LSM are figured for

the proportion detected*/

CLASS PathogenByType Article Sampling_Method EHMvsSBS;

/* Note that REML and GENMOD struggle with data that is too good a fit,

producing a complete or quasi-complete separation of the model.

Even if SAS produces output, parameter and error estimates cannot be trusted.

Therefore there is a formal process to identify the causes of this problem and

result in a robust model. See (Allison, 2001).

Here we document the various models we went through to arrive at the final

robust model reported in the text. To see the output from earlier models

remove comments around the relevant MODEL statement, and comment out any

other active MODEL statement*/

/* 1. "ideal model" perfect fit, usual errors, not viable */

/*

MODEL Detected_YesNo = EHMvsSBS Sampling_Method(EHMvsSBS ) Article PathogenByType EHMvsSBS*PathogenByType/ DIST=Binomial LINK=Logit ALPHA=0.05 type3;

*/

/* 2. remove article... finds a solution, but still clearly separated, */

/*

MODEL Detected_YesNo = EHMvsSBS Sampling_Method(EHMvsSBS ) PathogenByType EHMvsSBS*PathogenByType/ DIST=Binomial LINK=Logit ALPHA=0.05 type3;

*/

/*3. same as above, but now let's try removing the NS interaction */

/*This is the model reported in the text */

MODEL Detected_YesNo = EHMvsSBS Sampling_Method(EHMvsSBS ) PathogenByType / DIST=Binomial LINK=Logit ALPHA=0.05 type3;

/* Using GEE to include article produces the same result,

but involves additional assumptions, so the simpler analysis above is used */

/*

MODEL Detected_YesNo = EHMvsSBS Sampling_Method(EHMvsSBS ) PathogenByType / DIST=Binomial LINK=Logit ALPHA=0.05 type3;

repeated subject=article;

*/

lsmeans PathogenByType / ilink;

lsmeans EHMvsSBS / ilink;

lsmeans Sampling_Method(EHMvsSBS ) / ilink;

RUN;

/*Pathogen by Importance Analysis */

PROC GENMOD DATA=EHMSysRevMatch20240117 DESC;

/* The DESC sorting option ensures that the LSM are figured for

the proportion detected*/

CLASS PathogenByImportance Article Sampling_Method EHMvsSBS;

/* Note that REML and GENMOD struggle with data that is too good a fit,

producing a complete or quasi-complete separation of the model.

Even if SAS produces output, parameter and error estimates cannot be trusted.

Therefore there is a formal process to identify the causes of this problem and

result in a robust model. See (Allison, 2001).

Here we document the various models we went through to arrive at the final

robust model reported in the text. To see the output from earlier models

remove comments around the relevant MODEL statement, and comment out any

other active MODEL statement*/

/* 1. "ideal model" perfect fit, usual errors, not viable */

/*

MODEL Detected_YesNo = EHMvsSBS Sampling_Method(EHMvsSBS ) Article PathogenByImportance EHMvsSBS*PathogenByImportance/ DIST=Binomial LINK=Logit ALPHA=0.05 type3;

*/

/* 2. remove article... finds a solution, no evidence of model separation.

Interaction is significant, but shows evidence of quasi-complete separation

in that the parameter and error estimates are implausibly large

and may not be trustworthy. Confirmed by NS slices despite sigificance

of the interaction. */

/*

MODEL Detected_YesNo = EHMvsSBS Sampling_Method(EHMvsSBS ) PathogenByImportance EHMvsSBS*PathogenByImportance/ DIST=Binomial LINK=Logit ALPHA=0.05 type3;

lsmeans PathogenByImportance*EHMvsSBS / ilink;

Slice PathogenByImportance*EHMvsSBS;

*/

/*3. same as above, but now removing the separated interaction */

/*This is the model reported in the text */

MODEL Detected_YesNo = EHMvsSBS Sampling_Method(EHMvsSBS ) PathogenByImportance / DIST=Binomial LINK=Logit ALPHA=0.05 type3;

/* Using GEE to include article produces the same result,

but involves additional assumptions, so the simpler analysis above is used */

/*

MODEL Detected_YesNo = EHMvsSBS Sampling_Method(EHMvsSBS ) PathogenByImportance / DIST=Binomial LINK=Logit ALPHA=0.05 type3;

repeated subject=article;

*/

lsmeans PathogenByImportance / ilink;

lsmeans EHMvsSBS / ilink;

lsmeans Sampling_Method(EHMvsSBS ) / ilink;

RUN;
