# Supplementary material for: Do we still need a canary in the coal mine for laboratory animal facilities? A systematic review of environmental health monitoring versus soiled bedding sentinels
Source: PLoS One. 2024 Dec 5;19(12):e0311840. doi: 10.1371/journal.pone.0311840 (PMC11620448; doi:10.1371/journal.pone.0311840)
Supplement: Attachment — Submitted filename: Comments for Manuscript Number PONE-D-24-05570.pdf [file pone.0311840.s009.pdf]

Comments:

1. Great job and thank you for doing this work.

2. Page 3 line 61: *"Traditionally, rodent health monitoring programs have relied on sentinel rodents, that have an intact immune system and are not directly involved with experimental studies."*

Some programs use immunodeficient mice in certain areas or situations or rely on so called "provocation tests" to immunosuppress SBS to activate a dormant infection. Suggest this topic be expanded here or the discussion section given you do mention the use of nude mice (line 393).

3. Page 3 line 66: "*Sentinel animals are typically exposed at each cage-change, serially sampled, euthanized, and then replaced after 3-6 months [1]. Typically, there is one SBS cage per rack, or per side of a rack, both to give specificity as to the location of a pathogen and to increase sensitivity by avoiding dilution of the pathogen in too large of a pool of soiled bedding."*

First, note there are descriptions of live sentinel programs using mice for up to 12 months. See Lab Anim (NY). 2003 May;32(5):36-43.doi: 10.1038/laban0503-36. Second while you describe a "typical" program, it would be good to mention there are no agreed upon standards for SBS programs and now neither for non-animal alternatives discussed here. I agree with the overall assessment, but it is important to point out to the readers every comparison must be evaluated considering very specific context. Perhaps the discussion section is best to touch on this topic.

4. Page 4 line 106: "As a related advantage, unlike SBS, EHM can reveal both active and past infections from which a colony has recovered [15]."

I believe this statement to be incorrect. Overall, it really depends on testing frequency and the agent in question. SBS can detect active and past infections. For example, a parasitological assay such as anal tape test on a sentinel mouse can identify an active infection in the colony, and a serological assay can detect infections from which a colony may have recovered by the time a positive serological conversion is detected.

5. Page 4 line 107: *"Second, because the sensitivity of EHM comes from the sensitivity and specificity of PCR based assays, EHM avoids the complexity of using differing methods of detection for different pathogens and can detect even minute levels of pathogen genomic sequences in samples collected from the environment [15]."*

Given every assay has a detection limit it is possible minute amounts of pathogen genomic sequences that could not be detected by EHM (PCR), could in fact be multiplied in an exposed SBS allowing detection, albeit somewhat delayed. Correct?

6. Page 4 line 110: *"Third, EHM eliminates any animal welfare concerns as well as animal and labor costs inherent to a SBS program [22]."*

The statement makes it sound like there are no labor cost when using EHM, clearly not the case.

7. Are there any benefits to SBS over EHM that can be discussed here? How about the ability to identifying adventitious agents for which there is no PCR assay, or it is not included in the test panel? What happens if a wild mouse carrying unknown agents enters a vivarium and exposes and infects colony animals with agents not included in the EHM PCR panel? Biosecurity failures aside, how would

one find such agents? SBS might not find them either but there are examples in the literature that show this has happened.

8. Page 12, line 274: The acronym GEE appears twice in the manuscript, but it is not defined. Please do so.

9. I agree with the overall conclusions, but the manuscript seems a bit biased towards non-animal alternatives to SBS. It avoids any mention of positive attributes of SBS programs some of which I have mentioned before and perhaps should be included in the discussion section. I suggest you temper the tome to avoid alienation of the more traditional minded readers.

One item I thought was lacking in the evaluation of the literature comparing SBS with non-animal alternatives is the fact the assays used on SBS on those papers were not indicated in the supplemental materials. For example, the S1_Table_Pathogens does not specify which methods were used to detect each agent listed when using SBS. While non-animal alternatives rely solely on PCR, SBS can be tested using serological methods, PCR (skin, oral, feces, organs), bacterial cultures, traditional parasitological assays (scrape, floats, wet mounts), and histological evaluations. All these impacts, to a great degree, the detection rates and therefore the overall scoring to SBS programs. It has been shown, for example, that PCR on SBS sentinels is better than fecal float to detect some pinworms, and a combination of fecal PCR plus cecal examination is better than fecal PCR alone.

This is something I suggest should be discussed, perhaps as another factor that complicates these types of comparisons.
